# Spontaneous Rupture of Pyometra Causing Peritonitis in Elderly Female Diagnosed on Dynamic Transvaginal Ultrasound

**DOI:** 10.1155/2016/1738521

**Published:** 2016-02-18

**Authors:** Sharad M. Malvadkar, Madhuri S. Malvadkar, Shilpa V. Domkundwar, Shariq Mohd

**Affiliations:** Department of Radiodiagnosis, Grant Medical College and Sir JJ Group of Hospitals, Byculla, Mumbai 400008, India

## Abstract

Pyometra is collection of pus within the uterine cavity and is usually associated with underlying gynaecological malignancy or other benign causes. Spontaneous rupture of pyometra is a rare complication. We report a case of a 65-year-old female who presented with acute abdomen and was diagnosed with a ruptured uterus secondary to pyometra and consequent peritonitis on dynamic transvaginal sonography (TVS) which was later confirmed on contrast enhanced computed tomography (CECT). An emergency laparotomy was performed and about 800 cc of pus was drained from the peritoneal cavity. A rent was found in the anterior uterine wall and hence hysterectomy was performed. Histopathology revealed mixed inflammatory cell infiltrate with no evidence of malignancy. There are only 31 cases of ruptured pyometra reported till date, most of which were definitively diagnosed only on laparotomy. In only two of these cases the preoperative diagnosis was made on CECT. We report this case, as the correct and definitive diagnosis was made preoperatively on dynamic TVS. To our knowledge,* this is the first case report revealing spontaneous ruptured pyometra being diagnosed preoperatively on dynamic TVS*. This report is aimed at giving emphasis on the use of simple dynamic TVS for accurate diagnosis of rare spontaneous ruptured pyometra causing peritonitis.

## 1. Introduction

Pyometra is collection of pus in the uterine cavity with several aetiologies like malignancy of genital tract more commonly and sometimes benign lesions and other benign conditions. Clinical presentation of pyometra is a whitish discharge per vaginum and sometimes lower abdominal pain and bleeding per vaginum also. Spontaneous rupture of pyometra is a rare complication. Usually a definite diagnosis of spontaneous rupture pyometra was made only by laparotomy. This report is aimed at giving emphasis on the use of simple dynamic TVS for accurate diagnosis of rare spontaneous ruptured pyometra causing peritonitis.

## 2. Case History

A 65-year-old postmenopausal diabetic female presented to the emergency department with complaints of abdominal pain, fever, and distension along with white discharge per vaginum for seven days. Her vital parameters were stable except for mild tachycardia (pulse rate being 110 beats/min). Blood pressure was 110/70 mm Hg. Temperature was 100 degree F. Diffuse guarding, rigidity, and tenderness were present on abdominal palpation. On per speculum cervix was stenosed with minimal white discharge through external os. Per vaginal examination revealed retroverted normal sized uterus with free fornices.

Laboratory investigations revealed neutrophilic leukocytosis (white cell count of 14,800/cc with 66% neutrophils), elevated ESR of 32 mm/hour, and a low haemoglobin 10.7 gm/dL.

**Figure 1:**
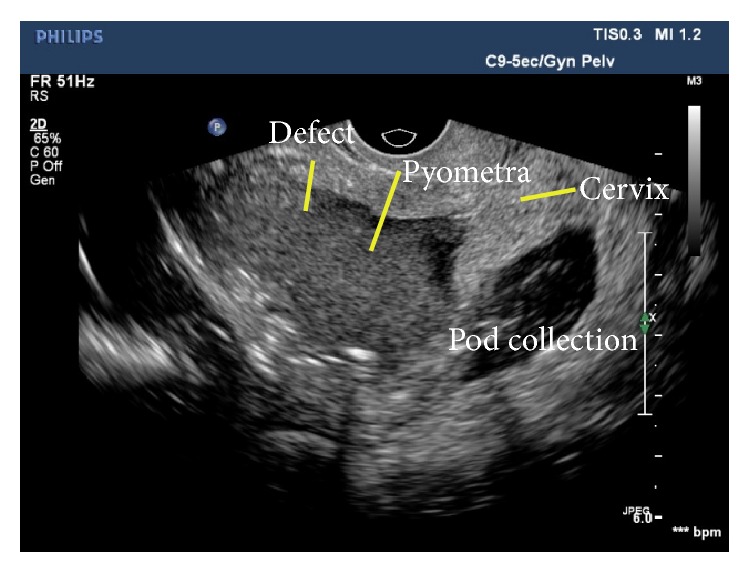
B mode TVS reveals large pyometra distending endometrial cavity with defect in anterior wall near fundus and collection in pouch of Douglas.

**Figure 2:**
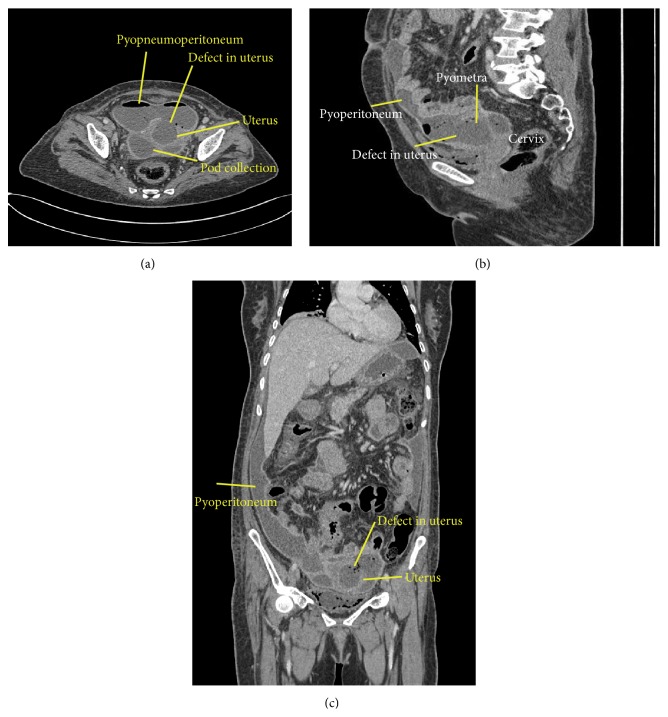
(a) Axial contrast enhanced CT in venous phase reveals pyometra communicating with peritoneal collection through defect in anterior wall of the uterus near fundus. Free nondependant air with smooth peritoneal enhancement suggesting pyopneumoperitoneum. (b) Sagittal contrast enhanced CT in venous phase reveals pyometra communicating with peritoneal collection through defect in anterior wall of the uterus near fundus. (c) Coronal contrast enhanced CT abdomen in venous phase reveals pyometra communicating with peritoneal collection through defect in the uterus near fundus.

First, a transabdominal ultrasound (USG) was performed which revealed moderate free fluid with thick echoes within the peritoneal cavity and mild collection within endometrial cavity. TVS was performed to look for a cause of endometrial collection, which revealed a collection with thick echoes within the uterine cavity suggestive of pyometra and a defect of size 2.0 × 2.0 cm in the anterior wall near fundus (Figure 1). Using a transvaginal probe gentle pressure was exerted over the cervix and uterus, which showed the real time movement of the endometrial collection through the defect into peritoneal collection with near complete emptying of the cavity. On releasing pressure, movement in the reverse direction was noticed with refilling of the endometrial cavity (See video in Supplementary Material available online at http://dx.doi.org/10.1155/2016/1738521). Based on these findings a diagnosis of spontaneous rupture of pyometra into peritoneal cavity was suggested. Emergency CECT abdomen and pelvis was performed which confirmed the pyometra, defect of 2.0 × 2.0 cm in the anterior wall of the uterus, and moderate peritoneal collection with multiple air pockets and smooth peritoneal enhancement suggesting pyeopneumoperitoneum with peritonitis (Figures 2(a), 2(b), and 2(c)).

**Figure 3:**
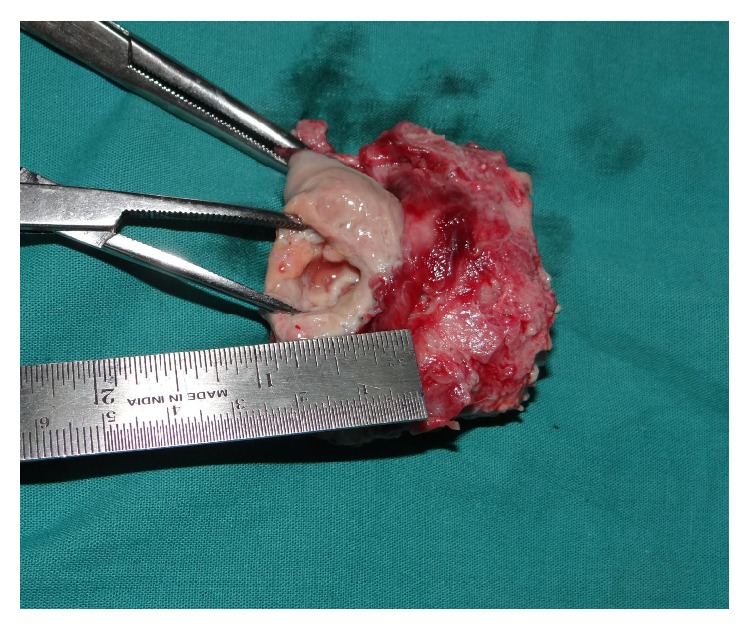
Postoperative surgical specimen showing 2 × 2 cm defect in the anterior wall near fundus of the uterus.

An emergency exploratory laparotomy was done. At laparotomy a defect of 2 × 2 cm was found in the anterior wall of the uterus and hence abdominal hysterectomy was performed (Figure 3). No evidence of any uterine or cervical malignancy was found. 800 cc of pus was drained from the peritoneal cavity followed by peritoneal lavage. Histopathology revealed haemorrhage along with fibrovascular tissue infiltrated with mixed inflammatory cell infiltrate comprising of polymorphs and a few lymphocytes confirming pyogenic infection. The patient was started on broad spectrum antibiotic and was discharged on 28th postoperative day without complication.

## 3. Discussion

Pyometra is defined as the collection of pus in the uterine cavity. The main cause of pyometra is cervical canal occlusion usually secondary to carcinoma cervix; however, other benign causes are endometrial polyp, leiomyoma, infection especially senile cervicitis, a forgotten intrauterine device, cervical occlusion after surgery, and radiation [1–3]. The usual presentation of pyometra is a whitish discharge per vaginum. Sometimes the patients may present with the clinical triad of abdominal pain, purulent vaginal discharge, and postmenopausal bleeding. However, more than 50% of all cases are asymptomatic [4].

Spontaneous rupture of pyometra is a rare complication, the incidence being 0.01–0.5% of all gynaecological patients and more common in postmenopausal females [5]. There are only 31 cases of ruptured pyometra reported till date [6–8].

Spontaneously perforated pyometra is difficult to diagnose preoperatively. Clinically it commonly mimics the symptoms of gastrointestinal tract diseases. Misdiagnoses are common and most frequent preoperative diagnosis is generalised peritonitis secondary to gastrointestinal perforation [9]. It is mentioned in prior case reports that, in most cases, a correct and definite diagnosis of spontaneous rupture pyometra was made only by laparotomy [8].

Preoperative diagnosis of perforated pyometra on CECT was made in only two cases in which CT suggested the diagnosis and surgical intervention was performed [10, 11]. Abdominal USG has high sensitivity in assessing pyometra, but it plays a limited role in the diagnosis of perforated pyometra because of its inability to demonstrate the uterine breach and the limited sonographic window available due to pneumoperitoneum [10]. However, this limitation can be overcome by TVS using a window of cervix and pyometra for detecting uterine defect and using dynamic TVS for demonstrating the real time movement of the endometrial collection through the defect into peritoneal collection.* There is no case report suggesting perforated pyometra being diagnosed preoperatively on dynamic TVS till date*. This report is aimed at giving emphasis on the use of simple dynamic TVS for accurate diagnosis of rare spontaneous ruptured pyometra causing peritonitis.* The advantage of USG over CT is accessibility, affordability, and no risk of radiation*. USG is readily available and cheaper compared to CT and hence helpful to patients of smaller institutions especially in developing countries.

In our case, the patient presented with symptoms of generalised peritonitis and TVS demonstrated the pyometra and a defect in the anterior wall of the uterus near fundus. Dynamic TVS showed real time movement of endometrial collection and peritoneal collection through defect in either direction further confirming diagnosis of perforated pyometra. The findings were confirmed on subsequent CECT abdomen, which revealed pyometra, uterine perforation in anterior wall near fundus, and pyeopneumoperitoneum.* To our knowledge this is the first such case report revealing spontaneous ruptured pyometra causing peritonitis being diagnosed preoperatively on dynamic TVS*.

The treatment of spontaneously ruptured pyometra is emergency laparotomy, peritoneal lavage and drainage, and hysterectomy.

## 4. Conclusion

Although spontaneous rupture of pyometra is rare, it should be kept in mind as a differential diagnosis in postmenopausal women presenting with acute abdomen and dynamic transvaginal sonography should be advised prior to laparotomy for definitive diagnosis. USG is more accessible and cheaper compared to CT and hence helpful in diagnosis of ruptured pyometra in patients from smaller institutions especially in developing countries. A carefully performed dynamic transvaginal sonography will give the accurate diagnosis so as to help with early intervention and proper treatment to reduce the associated morbidity and mortality.

## Supplementary Material

Video of dynamic transvaginal ultrasound reveals real time movement of endometrial collection through defect into peritoneal cavity on increasing transvaginal probe pressure on cervix and refilling of endometrial cavity by reverse movement of peritoneal collection into endometrial cavity after release of pressure, confirming diagnosis of spontaneous rupture of pyometra.
